# Case Report: Diode laser fenestration for treating conchal malformation in a miniature pony

**DOI:** 10.3389/fvets.2026.1843048

**Published:** 2026-10-22

**Authors:** Patricia Trummer-Schug, Orietta Rother, Olivia Mariel Grünzweil, Michaela Gumpenberger, Agnieszka Florczyk

**Affiliations:** 1 Department for Small Animals and Horses, Center for Equine Health and Research, University of Veterinary Medicine Vienna, Vienna, Austria; 2 Department for Small Animals and Horses, Clinical Unit of Diagnostic Imaging, University of Veterinary Medicine Vienna, Vienna, Austria

**Keywords:** case report, computed tomography, diode laser fenestration, miniature pony, sino-nasal obstruction, conchal malformation, sinusitis, standing surgery

## Abstract

**Background:**

Chronic unilateral nasal discharge in miniature horses is often caused by dental disease, infection, or anatomical abnormalities. Miniature breeds may be predisposed to sino-nasal obstruction due to disproportionately large teeth and retained juvenile cranial features, which can impair sinus drainage. Congenital or developmental sino-nasal malformations are rarely reported in equids.

**Case description:**

A one-year-old Shetland pony filly presented with an eight-week history of unilateral nasal discharge unresponsive to antimicrobial therapy. Endoscopy revealed a displaced dorsal concha overlapping a hypoplastic ventral concha, with malformed drainage pathways. Computed tomography confirmed marked right-sided sino-nasal anatomical abnormalities, including dorsal concha enlargement, ventral concha hypoplasia, and septal deviation.

**Clinical findings:**

The filly was bright and alert, with right-sided purulent nasal discharge. Bloodwork revealed mild anemia, lymphopenia, and elevated inflammatory markers. Guttural pouch lavage grew *Streptococcus equi* subsp. zooepidemicus, while dental examination was unremarkable.

**Treatment and outcome:**

Conservative therapy with meloxicam and doxycycline was unsuccessful. Under standing sedation, diode laser fenestration of the dorsal concha was performed, creating a 3 cm drainage window to restore sinus communication and allow transnasal lavage. Postoperative endoscopy confirmed a patent fenestration with reduced mucosal inflammation. The filly showed rapid clinical improvement and was discharged 6 days later. At a follow-up examination 14 months after surgery, the pony was free of purulent nasal discharge and the owners reported improved activity and quality of life. Endoscopy showed that the fenestration had closed by scar formation, while the nasomaxillary aperture was accessible and the drainage angle appeared normal, consistent with continued growth of the skull.

**Conclusion:**

Congenital or developmental conchal malformations should be considered in miniature horses with chronic unilateral nasal discharge. Advanced imaging, particularly CT, is crucial for diagnosis and surgical planning. Transendoscopic diode laser fenestration is a minimally invasive option to restore sinus drainage and to enable repeated lavage. In this growing pony the fenestration closed over time, while physiological drainage was re-established as the sino-nasal anatomy matured, suggesting that temporary drainage may be sufficient to interrupt the cycle of chronic sinusitis in selected cases.

## Introduction

Chronic or recurrent nasal discharge in equids is a common clinical problem that may result from a wide range of underlying causes, including dental disease or infectious processes. Less common causes of secondary sinusitis include ethmoid hematomas and paranasal cysts, while congenital or developmental anatomical abnormalities of the sino-nasal regions are considered rare but clinically relevant triggers (1–3). Miniature horses, such as Shetland ponies, are thought to have a higher prevalence of dental and upper respiratory tract disorders due to their small stature and disproportionately large teeth which do not scale with the skull and body size (4, 5). To date, only a small number of idiopathic sino-nasal obstruction cases, mostly in young horses and ponies, have been reported, typically as single cases or small series (6–8). Furthermore, miniature horses often retain juvenile cranial characteristics that might contribute to the problem (9). The paranasal sinus system in Shetland ponies resembles that of large horses, with all seven compartments and a similar bilateral rostral and caudal arrangement. The rostral compartment comprises the rostral maxillary sinus and the ventral conchal sinus; the caudal compartment comprises the caudal maxillary sinus, the frontal sinus, the sphenopalatine sinus, the dorsal conchal sinus and the middle conchal sinus (10). However, identifying the sino-nasal communication channels was challenging and inconsistent due to anatomical complexity (10). These anatomical differences may predispose them to congenital or developmental malformations of the nasal passages and paranasal sinuses. Thus, normal mucociliary clearance is impaired which leads to chronic sinusitis.

Definitive diagnosis necessitates a comprehensive clinical and dental examination as well as diagnostic radiography. A thorough transnasal endoscopy of the nasal passages, paranasal sinuses, and upper respiratory tract structures, including the guttural pouches, is also crucial. In instances where clinical signs persist despite appropriate medical intervention, advanced imaging techniques, most notably computed tomography (CT), are indispensable for detecting underlying structural pathology and formulating an effective therapeutic strategy (2, 11–13). Transnasal treatment of equine sinusitis using endoscopic lavage and debridement has been reported in cases where spontaneous sino-nasal fistulation provides access to the sinus cavities (2). In chronic cases, progressive remodeling of the conchal and sinus structures may alter or narrow communication pathways, which can make endoscopic access technically more challenging, although transnasal laser techniques remain a viable treatment option in many patients (8, 14, 15). Experimental studies by Morello and Parente and by Kološ et al. demonstrated that endoscopically guided diode laser fenestration of the dorsal concha can create a controlled sino-nasal fistulation, enabling access to the caudal paranasal sinuses (14, 16). Clinical applications of this technique have since been described, showing that diode laser fenestration of the dorsal or ventral concha allows therapeutic exploration and treatment of otherwise inaccessible sinus compartments in selected cases (16–19). Data on the long-term patency of such fenestrations are, however, limited. In addition, other minimally invasive approaches have been described to improve the sinus drainage, for example surgical enlargement of the nasomaxillary aperture combined with transnasal conchotomy, transnasal conchotomy alone and widening of the nasomaxillary aperture with a Foley catheter (15, 18, 20).

This case report presents a one-year-old miniature Shetland pony filly with chronic unilateral nasal discharge associated with a congenital or developmental malformation of the right nasal conchal structures and secondary sinusitis. It describes the diagnosis and management of dorsal conchal malformation in a miniature pony, with a focus on the use of computed tomography (CT) and transendoscopic diode laser fenestration. The case highlights the diagnostic utility of CT and endoscopy for identifying complex sino-nasal abnormalities. In addition, it demonstrates that diode laser fenestration of the dorsal concha is a minimally invasive and effective technique for restoring sinus drainage and resolving chronic sino-nasal inflammation in small equids. It further reports the clinical and endoscopic findings 14 months after surgery, providing long-term follow-up data that are currently lacking for this technique in miniature equids.

## Case presentation

### Signalment and case history

The one-year-old mini-Shetland pony filly was presented to the Equine Clinic of the University of Veterinary Medicine Vienna in July 2025 due to unilateral nasal discharge. The filly had been exhibiting nasal discharge for 8 weeks prior to submission. Fever had also been noted. Conservative treatment prior to referral with trimethoprim at 5 mg/kg and sulfadiazine at 25 mg/kg twice daily and acetylcysteine at 10 mg/kg once daily was ineffective. Additionally, no relevant familial or genetic history was known.

### Clinical findings

The filly (60 kg) was bright, alert, and in good nutritional condition at presentation. Clinical examination revealed bilateral conjunctival hyperemia, pale oral mucous membranes, mildly increased vesicular breath sounds and mild, non-painful bilateral mandibular lymphadenopathy. No cough, facial swelling, nasal deformity, or epiphora was observed.

Upper airway endoscopy using a 9.2 mm × 1.9 m enteroscope (SIF-Q180, Olympus, Japan) revealed mild nasal discharge in the right nasal passage, left-sided mucosal edema, moderate retropharyngeal lymphadenopathy on the left side and mild dynamic tracheal collapse. Guttural pouch lavage was performed to exclude *Streptococcus equi* subsp. *equi* as a potential primary cause of the nasal discharge and the previously described fever. The lavage sample grew *Streptococcus equi* subsp. *zooepidemicus* (qPCR for *S. equi* subsp. *equi* negative), and bloodwork showed mild anemia, lymphopenia, and elevated GLDH, CK, and serum amyloid A.

Dental examination via endoscope (Dental Telescope 66490 CF, Tele Pack Vet X LED RP 100, KARL STORZ SE & Co KG, Germany) was age-appropriate and unremarkable.

Transnasal sinus endoscopy was performed using a 5.4 mm and 1.1 m long gastroscope (EG-1690-K Videogastroskop, Pentax Europe GmbH, Germany). In the caudal third of the right nasal passage, in the region of the dorsal conchal recess, the dorsal concha was dorsally displaced and overlapped a hypoplastic ventral concha. Purulent material was present in the middle meatus between the dorsal and ventral conchae. The drainage angle was malformed.

Differential diagnoses included dental disease, primary sinusitis, and guttural pouch infection.

### Radiographic and computed tomographic findings

Standard radiographic projections of the head were obtained, comprising laterolateral, dorsoventral, and oblique projections of the maxilla (L30-35°D-LVO), no special or complementary projections were performed. No fluid line was visible within the paranasal sinuses. Additionally, a computed tomographic examination was performed. Radiographic and computed tomographic examinations revealed age-appropriate structures. The most relevant findings are shown in Figures 1, 2.

**Figure 1:**
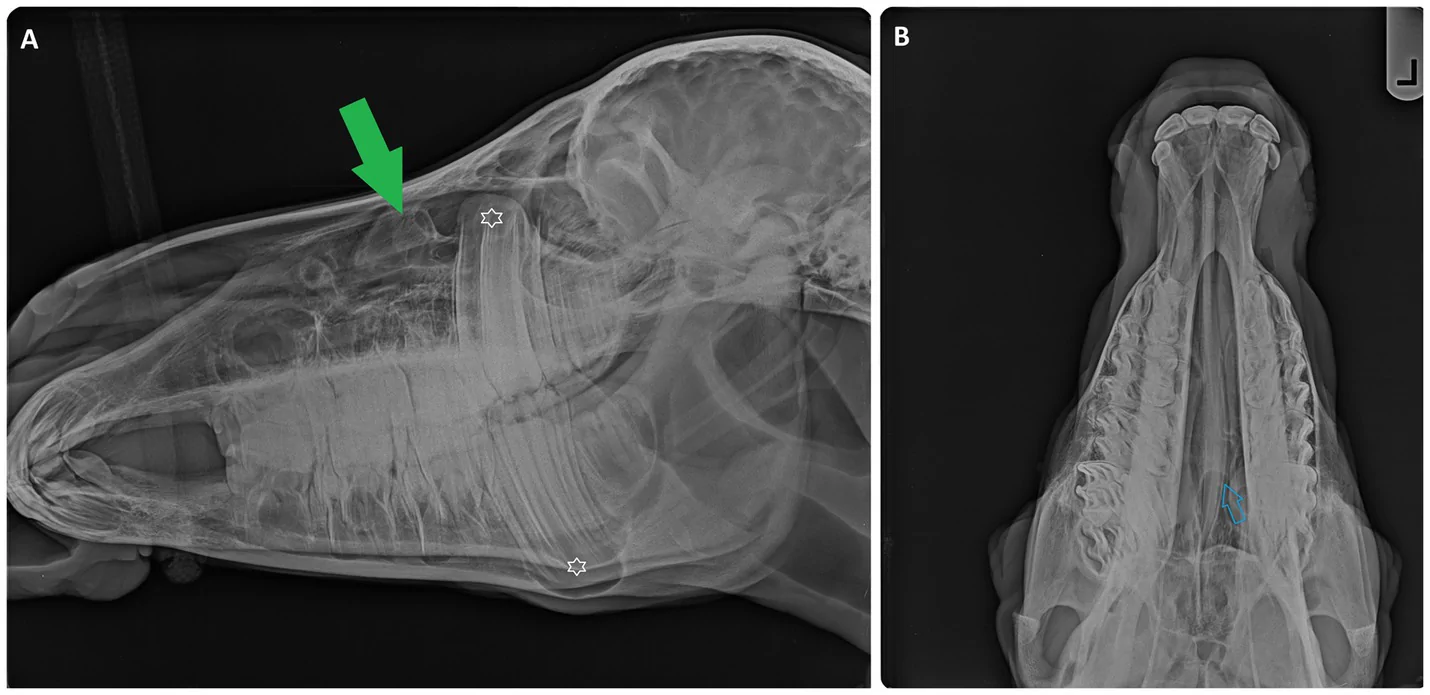
Laterolateral **(A)** and dorsoventral (frontonasal) **(B)** skull radiograph. **(A)** A marked disproportion between tooth size and number relative to overall skull dimensions is evident. A questionable area of increased soft tissue opacity is observed within the dorsal conchal bulla, suggestive of mild sinusitis (indicated by green arrow). The developing first molars are prominently visible (marked with white asterisks) in both the maxilla and mandible. The second molar tooth buds are identifiable, whereas the third molars are not yet radiographically apparent. **(B)** A marked disproportion between tooth size and number relative to overall skull dimensions is evident. Additionally, a deviation of the nasal septum toward the left side is observed (indicated by blue arrow).

**Figure 2:**
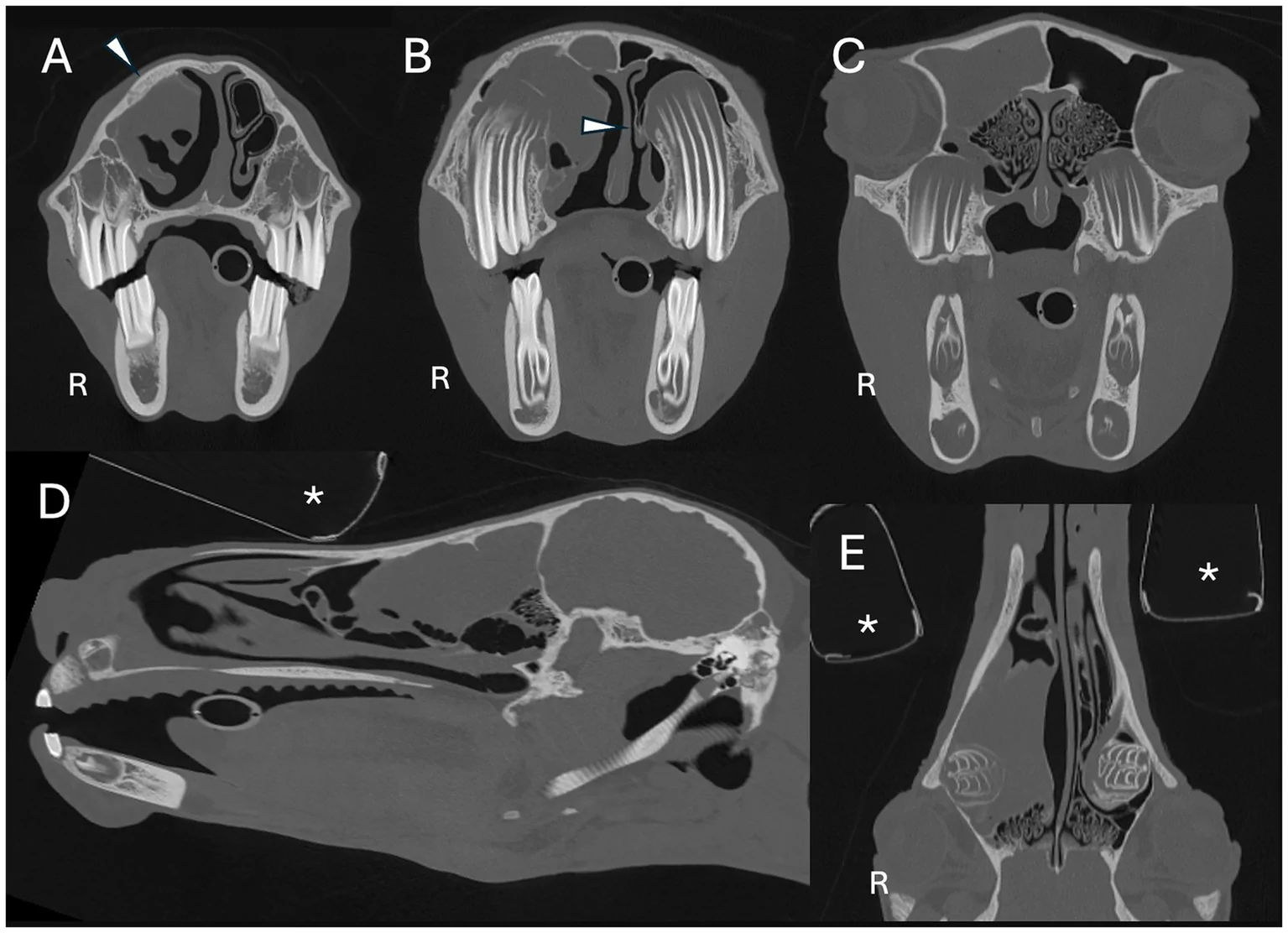
CT images in a bone window in transverse **(A–C)**, paramedian sagittal **(D)** and coronal **(E)** reconstructions of a one-year-old Shetland pony filly. **(A)** is at the level of the cranial half of 08, **(B)** at the level of 09, and **(C)** at the level of the ethmoid bone. **(D,E)** Highlight the center of the nasal cavities. A marked asymmetry between the right and left sides of the head is clearly visible, including a midline shift of the nasal septum and bulging of the dorsum nasi (arrowheads). More than half of the nasal cavity on the right side is filled with soft-tissue-dense material and thickened mucosa, while the normal anatomical structures are lost. * positioning device.

### Diagnosis

The filly was diagnosed with congenital or developmental sino-nasal abnormalities resulting in chronic nasal discharge.

### Treatment plan

The filly was treated for 10 days with meloxicam (Rheumocam® 15 mg/mL, Chanelle Pharmaceuticals Manufacturing Ltd., Ireland) at 0.6 mg/kg once daily per os. Additionally, she received doxycycline at 10 mg/kg (Pulmodox® 500 mg/g, Lavet Pharmaceuticals Ltd., Hungary) twice daily for 12 days per os. Conservative treatment was initiated based on clinical findings in the left guttural pouch and the detection of *Streptococcus equi* subsp. *zooepidemicus*. The filly was kept in isolation until *Streptococcus equi* subsp. *equi* was ruled out by qPCR testing.

Due to the anatomical abnormalities and insufficient sinus drainage, diode laser fenestration of the dorsal concha was elected as the preferred treatment approach.

### Surgical intervention

Therapeutic laser surgery was performed under standing sedation and local anesthesia (splash block) using a diode laser to fenestrate the dorsal concha. This created a drainage window into the middle and ventral meatus, with the goal of improving airflow and facilitating effective drainage from the affected sinuses. The head was suspended in a head sling at the level of the withers. The same 5.4 mm × 1.1 m videogastroscope with a 2 mm working channel (EG-1690-K Videogastroskop, Pentax Europe GmbH, Germany) that had been used for the diagnostic sinus endoscopy was introduced through the right (ipsilateral) nostril and advanced through the common nasal meatus toward the dorsal concha. The laser fiber was angled dorsolaterally rather than advanced in a straight-on approach, in order to reach the fenestration site within the confined anatomical space. The nasal mucosa was not decongested prior to the procedure.

The extent of the fenestration was planned on the preoperative CT images of the affected side. It corresponded to the transverse levels of the maxillary cheek teeth 508 to 109, the region in which the dorsal concha overlapped the ventral concha.

The corresponding CT reconstructions and the planned site of fenestration are shown in Figure 3.

**Figure 3:**
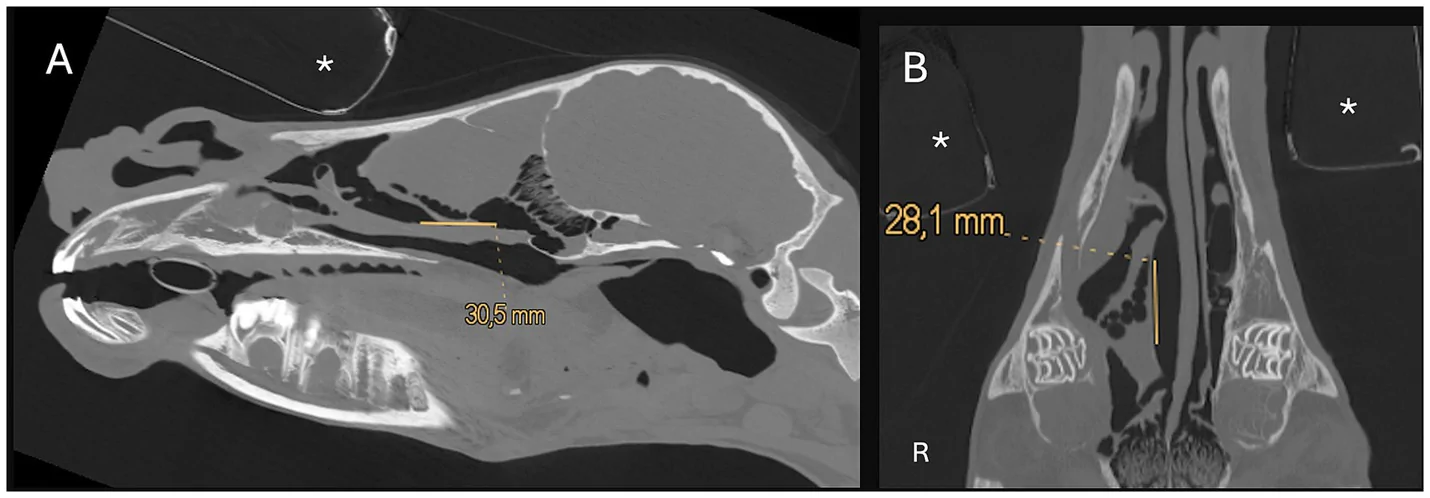
CT images (bone window), paramedian sagittal **(A)** and coronal **(B)** reconstructions. The orange line marks the site of laser fenestration of the dorsal nasal concha and indicates its estimated length. In **(A)**, the center of the nasal cavity at the level of the middle nasal meatus is highlighted. In **(B)**, the levels of cheek teeth 109 and 110 are indicated. *Positioning device.

The pony received intravenously an initial sedation of 0.012 mg/kg detomidine hydrochloride (Domidine® 10 mg/mL, Eurovet Animal Health B.V., Netherlands) and 0.012 mg/kg butorphanol tartrate (Butomidor® 10 mg/mL, VetViva Richter GmbH, Austria). This was followed by a constant rate infusion delivering 0.012 mg/kg of both detomidine hydrochloride and butorphanol tartrate diluted in 1 L of saline (B. Braun, Germany) to maintain sedation throughout the procedure. Additionally, she received flunixin meglumine (Niglumine® 50 mg/mL, Laboratorios Calier S.A., Spain) at a dose of 1.1 mg/kg IV for analgesia and anti-inflammatory purposes.

Before surgery, an endoscopically guided splash block was performed using lidocaine hydrochloride at a dose of 2.67 mg/kg combined with epinephrine at 0.67 μg/kg (Xylanaest® 2% mit Epinephrin 1:200,000; Gebro Pharma GmbH, Austria) via the working channel of the endoscope (5.4 mm, EG-1690-K Videogastroskop, Pentax Europe GmbH, Germany). The 600 μm diode laser fiber was then introduced through the same working channel. An 810 nm diode laser (MedArt 426, Asah Medico A/S, Hvidovre, Denmark) was used in contact mode at a power setting of 15 watts in continuous wave mode, delivering a total energy of 600 joules, which corresponds to approximately 40 s of laser emission, to achieve effective fenestration of the dorsal concha while minimizing thermal damage to surrounding tissues. The incision was made in a single continuous pass. It started at the caudal end of the overlapping portion of the dorsal concha, corresponding to the border between the middle and the caudal third of the concha and extended approximately 3 cm rostrally, as measured against the depth markings on the endoscope. The resulting fenestration was slit-shaped and slightly oval, the opening having widened as a result of the thermal effect of the laser. Smoke development did not impair visualization during cutting. Once the incision was completed, the laser was stopped and the smoke was allowed to clear before the fenestration was evaluated endoscopically. Hemorrhage was minimal throughout the procedure. The total procedure time, including preparation, was approximately 15 min. A moderate quantity of inspissated and fluid purulent material was evacuated from the sinus system intraoperatively. Following fenestration, transnasal sinus endoscopy was performed, allowing direct visualization and inspection of the sinus system. Within the maxillary sinus, the reserve crown of tooth 109 was prominently visible. The mucous membranes appeared moderately to markedly inflamed. The affected sinus was thoroughly flushed via high pressure transendoscopic lavage (OFP-2, Olympus Europa SE & CO. KG, Germany), using a total of 3 liters of sterile saline (NaCl 0.9%, Kochsalzlösung für Spülzwecke, Fresenius Kabi Austria GmbH, Austria) to remove inspissated material, debris, and inflammatory fluid.

### Outcome and follow-up

Postoperatively, the pony showed marked clinical improvement, including increased appetite, improved general demeanor and a noticeable reduction in nasal discharge. An additional dose of flunixin meglumine (1.1 mg/kg, IV) was administered 12 h after the procedure, followed by a course of phenylbutazone at 2.2 mg/kg PO twice daily for 3 days to manage postoperative inflammation and discomfort. No postoperative antibiotics were administered, consistent with our strict antimicrobial stewardship policy, the adequate restoration of sinus drainage, and the possibility of repeated sinus lavage.

Transnasal sinus endoscopy was performed on postoperative days two and five. These examinations confirmed a patent fenestration in the wall of the dorsal concha with continued access to the sinus system. The size of the opening stayed the same. The sinus was flushed with 1 L of sterile saline (NaCl 0.9%, Kochsalzlösung für Spülzwecke, Fresenius Kabi Austria GmbH, Austria) during each follow-up. Mucosal inflammation was visibly reduced and no further accumulation of purulent material was noted.

Serum creatinine was rechecked postoperatively to monitor renal function; other hematological and biochemical parameters were not repeated.

By postoperative day six, the filly was bright and clinically stable with only minimal serous nasal discharge. She was eating well and was discharged without the need for further treatment. The filly remained free of clinical signs at a four-month telephone follow-up with the owners. The owners reported a marked improvement in the pony’s energy level and quality of life following treatment. A re-examination was recommended 6 months postoperatively. However, the pony was not presented at that time. A follow-up examination was eventually performed 14 months after surgery. The pony was in good general condition, with mild bilateral serous nasal discharge, mild facial asymmetry and mild, non-painful swellings over both the maxillary and mandibular cheek teeth, consistent with age-appropriate eruption bumps. Transnasal endoscopy (FIVE S 5.3/2.2 mm × 65 cm, KARL STORZ SE & Co KG, Germany) was performed on both sides. Apart from mild mucous discharge and polypoid changes in the middle meatus at the level of the ventral conchal recess, the left nasal passage was unremarkable. The right nasal passage revealed mild mucous discharge along the entire length of the ventral, middle and common nasal meatus. Scar tissue had formed at the site of the laser fenestration, and the fenestration into the dorsal conchal sinus was no longer patent. However, the region of the nasomaxillary aperture was accessible and the drainage angle appeared normal. The previously overlapping portion of the dorsal concha was no longer visible (Figure 4).

**Figure 4:**
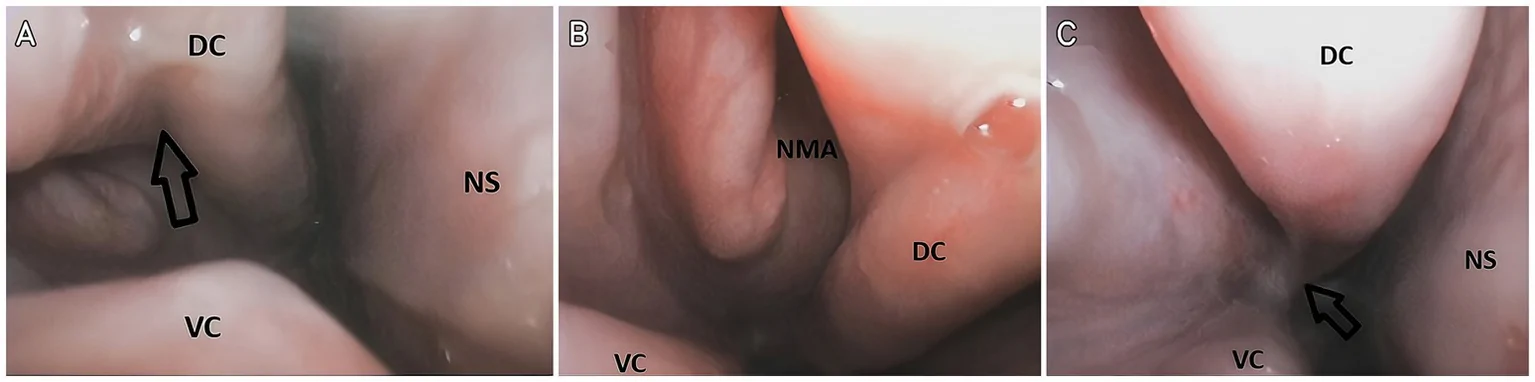
Transnasal endoscopic images of the right nasal passage at the 14-month follow-up, with the endoscope positioned in the middle nasal meatus. **(A)** Overview of the former fenestration site: scar tissue has formed at the site of the previous laser fenestration of the dorsal concha (black arrow) and the fenestration was no longer patent on endoscopic examination. **(B)** Entry to the nasomaxillary aperture. **(C)** A physiological drainage angle (black arrow). Mild mucous discharge is visible in **(A,B)**. DC, dorsal concha; VC, ventral concha; NS, nasal septum; NMA, nasomaxillary aperture.

Based on the localized nature of the sino-nasal malformation, successful restoration of sinus drainage, and the absence of recurrent purulent discharge 14 months after surgery, the prognosis for long-term resolution of clinical signs was considered favorable.

## Discussion

Abnormal sino-nasal anatomy is rarely described in horses. A limited number of studies have addressed this topic, particularly in miniature horses and ponies. Idiopathic sino-nasal obstruction has been documented in a few publications. This condition predominantly affects younger animals, which suggests a possible developmental origin (6–8). A breed predisposition may also be considered (7, 8). Furthermore, disproportionately large reserve crowns relative to skull size could contribute to impaired sino-nasal drainage. In the present case, the nasomaxillary aperture could not be visualized endoscopically prior to surgery. However, given the presence of nasal discharge, it is likely that complete obstruction was not present. Vlaminck et al. reported a case characterized by complete obstruction of all sino-nasal communication pathways, where the horse exhibited pronounced clinical signs including respiratory distress and open-mouth breathing (8). In the present case the filly was bright and alert and showed no signs of dyspnea, which further indicates that there was still a communication between the sinus system and the nasal cavity.

Initial conservative management with non-steroidal anti-inflammatory drugs (NSAIDs) and antimicrobials was unsuccessful. This proved to be consistent with the findings of Vlaminck et al. whose similar therapeutic approach failed to resolve clinical signs (8). In the current case, an accumulation of fluid produced by the respiratory mucosa within the sinus system was observed. CT revealed a visible deformation of the skull, with asymmetrically enlarged nasal cavities and paranasal sinuses on the right side and a marked deviation of the caudal nasal septum to the left. Similar findings were reported in a case series by Vlaminck et al. suggesting that chronic intranasal pressure from retained fluid may contribute to these structural changes (8). CT is widely used for the diagnosis of equine dental and sino-nasal disorders because it provides true three-dimensional imaging and eliminates the superimposition of adjacent structures (11, 21–25). In contrast, conventional radiography is limited by overlapping anatomy, which can obscure clinically relevant abnormalities. Recent work has shown that dedicated complementary projections, such as an offset dorsoventral or a lateral 75° dorsal-lateral ventral oblique view, can substantially improve radiographic visualization and identification of the nasal conchal bullae and conchal sinuses (26, 27). In the present case, the radiographs demonstrated a marked disproportion between tooth size and number relative to the overall skull dimensions, along with a focal increase in soft tissue opacity within the dorsal conchal bulla suggestive of mild sinusitis, and a deviation of the nasal septum to the left. Although these findings raised suspicion for underlying developmental and sino-nasal pathology, the radiographic interpretation was restricted by structural superimposition, highlighting the added value of CT in fully characterizing the abnormalities.

Treatment in the current case consisted of diode laser fenestration of the dorsal concha performed under standing sedation. While some previously published cases employed more invasive surgical techniques to restore sino-nasal drainage (8, 15), less invasive methods have also been described. These include transendoscopic laser fenestration (14, 16–18), endoscopically guided transnasal conchotomy (15, 18), and widening of the nasomaxillary aperture using a Foley catheter (18, 20). However, none of the less invasive techniques has been described in a miniature horse or pony.

This case demonstrates that a minimally invasive, transendoscopic diode laser approach can be an effective alternative for re-establishing physiological sino-nasal drainage in selected patients. The precise location for fenestration was determined based on preoperative CT findings, taking into account the anatomical constraints and accessibility in a standing, sedated pony. In this particular case, the ventral concha was markedly malformed and compromised, especially in the caudal region, precluding its use as a fenestration site. Additionally, in young equids, large reserve crowns further limit the feasibility of safely accessing the ventral conchal sinus (VCS), making dorsal conchal approaches more practical and targeted.

At the follow-up examination 14 months after surgery, the fenestration was no longer patent and had healed by scar formation. This was not unexpected, as mucosal defects in the equine nasal cavity heal by secondary intention. Importantly, closure of the fenestration was not associated with recurrence of purulent nasal discharge. Continued growth of the skull between 1 and 2 years of age appears to have changed the sino-nasal anatomy accordingly: the drainage angle, which had been malformed at presentation, was now normally developed and the nasomaxillary aperture, which could not be visualized endoscopically before surgery, was readily accessible. The fenestration of the dorsal concha was therefore necessary at the time of presentation to establish drainage and to allow repeated lavage, whereas at the follow-up examination physiological drainage was functioning well without it.

The remaining findings at the 14-month examination were considered clinically insignificant. Mild bilateral seromucous nasal discharge can occur intermittently in otherwise healthy horses. On the left side, polypoid changes were noted in the middle meatus at the level of the ventral conchal recess. As the pony showed no clinical signs referable to the left nasal passage, these changes were interpreted as an incidental finding. A mild contralateral manifestation of the underlying sino-nasal condition can, however, not be excluded and monitoring was recommended. The non-painful swellings over the maxillary and mandibular cheek teeth were consistent with eruption bumps and therefore age-appropriate in a two-year-old pony. The mild facial asymmetry had already been documented on the preoperative computed tomographic examination and is attributed to the underlying sino-nasal malformation rather than to the surgical procedure.

This case report has several limitations. As a single-case observation, the findings may not be generalizable to all miniature horses or ponies with sino-nasal malformations. The precise contribution of prior medical therapy to the favorable outcome cannot be fully determined. Additionally, no intraoperative or early postoperative endoscopic images are available, as the corresponding data were inadvertently lost during archiving. The dimensions of the fenestration are therefore documented only by the intraoperative measurement. At the follow-up examination, no repeat radiographic or computed tomographic imaging was performed and a different endoscope was used than at the initial presentation, which limits the direct comparability of the endoscopic views. In addition, histopathology was not performed, as imaging and endoscopy revealed no discrete mass suggestive of a proliferative lesion; a contributing inflammatory or proliferative component therefore cannot be fully ruled out. Future studies with larger case series are needed to confirm the efficacy, safety, and optimal parameters of diode laser fenestration in this population.

## Conclusion

Congenital or developmental malformations of the nasal conchal structures should be considered in young miniature horses presenting with chronic unilateral nasal discharge. In this case, CT and endoscopy were essential for characterizing the underlying sino-nasal abnormality and planning a targeted treatment. Transendoscopic diode laser fenestration of the dorsal concha successfully restored sinus drainage and led to rapid clinical improvement that was sustained 14 months after surgery, although at that time drainage occurred through the nasomaxillary aperture and the fenestration itself had closed by scar formation. This minimally invasive technique represents a useful therapeutic option for selected miniature equids with complex sino-nasal obstruction.

## Data Availability

The raw data supporting the conclusions of this article will be made available by the authors, without undue reservation.
